# Tricuspid annular plane systolic excursion (TAPSE) revisited using CMR

**DOI:** 10.1186/1532-429X-14-S1-P299

**Published:** 2012-02-01

**Authors:** Srinivas L Naik, Jeffrey J Rodriguez, Nishant Kalra, Vincent L Sorrell

**Affiliations:** 1 Electrical and Computer Engineering, University of Arizona, Tucson, AZ, USA; 2 Department of Cardiology, Sarver Heart Center University of Arizona, Tucson, AZ, USA; 3 Division of Cardiovascular Medicine and Gill Heart Institute., University of Kentucky, Lexington, KY, USA

## Summary

This observational pilot project was performed as background to eventually create a rapid, automated and accurate assessment of RV systolic function in variable clinical subgroups. We propose new parameters that characterize the global systolic function of the right ventricle with a simple linear measurement.

## Background

The tricuspid annular plane systolic excursion (TAPSE), which has been used for over a quarter century as a quick estimate of RV systolic function, was revisited using CMR. It shows good correlation with invasive hemodynamics. MRI is the current gold standard to assess the volumes and anatomy of the heart and we can measure TAPSE more precisely with cardiac MRI to accurately detect any current problems TAPSE measurements may suffer from.

## Methods

We studied 61 patients (32 female; mean age 46 and std. dev. 22yrs) with adequate, high quality, clinical CMR scans using a conventional 1.5T GE scanner were studied. In addition to simulating the echo-TAPSE parameter, additional semi-automated CMR parameters were obtained from manually identified phasic anatomic landmarks that honored the trajectory of the tricuspid annulus (TA) and right ventricle apex (RVA):

1. TAPSE -Distance traveled by the TA along the direction of the fixed line joining the TA and RVA at end-diastole (simulates echo measure).

2. Δ TA-RVA - Difference between lengths of line segments joining the (RVA) and TA at end-diastole and end-systole (recognizes cardiac translation).

3. TA Excursion (TAE) - Euclidean distance traveled by the TA from end-diastole to end-systole (measures just the TA motion).

The correlation of these parameters with conventional RVEF was obtained using linear regression analysis, and a p-value less than 0.05 was considered significant.

## Results

In general, there is a weak correlation between TAPSE and RVEF and a moderate correlation using the newer parameters of Δ TA-RVA and TAE. In clinically relevant subgroups such as RV pressure overload (RVPO) and RV volume overload (RVVO), there are statistically significant differences between the traditional and conventional parameters (see Table 1).

**Table 1:** Correlation coefficients for various CMR parameters and patient subgroups. (* p < 0.05)

Sugroups (N)	TAPSE	ΔTA-RVA	TAE
All Patients (61)	0.15	0.53*	0.51*
Normal RV (43)	0.02	0.24	0.15
Abnormal RV (18)	-0.32	0.54*	0.42*
RVVO (5)	-0.06	0.92*	-0.16
Non-RVVO (56)	0.13	0.43*	0.42
RVPO (4)	-0.54	0.001	0.70
Non-RVPO (57)	0.16	0.56*	0.51*

## Conclusions

In this small pilot project, CMR simulation of echo-TAPSE is a very poor marker of RVEF (r = 0.15), but this correlation can be significantly improved using the rapid, semi-automated Δ TA-RVA or TAE method. Very importantly, the choice of parameter has diametrically opposed influences on RVVO and RVPO sub-groups (Δ TA-RVA in RVVO, r = 0.92; TAE in RVPO, r = 0.70). These findings suggest variable alteration of longitudinal RV myocardial contraction and warrants further investigation.

## Funding

A portion of Dr. Sorrell's support for this project was provided by the Allan C. Hudson and Helen Lovaas Endowed Chair of Cardiac Imaging.

**Figure 1:**
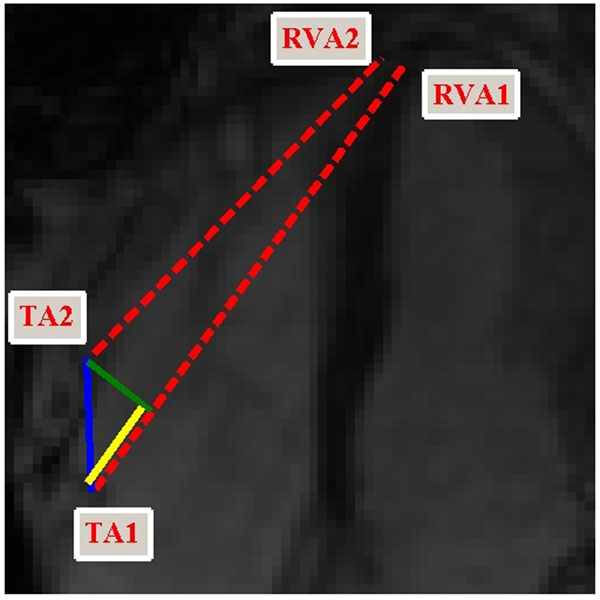
Calculation of proposed parameters demonstrated on end-diastole and end-systole frames for this particular image set. The red dotted lines show line segments joining TA1 and TA2 with RVA1 and RVA2, respectively, and Δ TA-RVA is the difference between these two segments lengths. TAE is the length of the blue line segment joining TA1 and TA2. TAPSE is the projection (in yellow) of the blue segment onto the line joining TA1 and RVA1.

